# *RERG* suppresses cell proliferation, migration and angiogenesis through ERK/NF-κB signaling pathway in nasopharyngeal carcinoma

**DOI:** 10.1186/s13046-017-0554-9

**Published:** 2017-06-28

**Authors:** Weilin Zhao, Ning Ma, Shumin Wang, Yingxi Mo, Zhe Zhang, Guangwu Huang, Kaoru Midorikawa, Yusuke Hiraku, Shinji Oikawa, Mariko Murata, Kazuhiko Takeuchi

**Affiliations:** 1 Department of Environmental and Molecular Medicine, Mie University Graduate School of Medicine, 2-174, Edobashi, Tsu, Mie 514-8507 Japan; 2 Department of Otorhinolaryngology - Head and Neck Surgery, Mie University Graduate School of Medicine, 2-174, Edobashi, Tsu, Mie 514-8507 Japan; 3 Department of Otolaryngology Head and Neck Surgery, First Affiliated Hospital of Guangxi Medical University, Nanning, Guangxi China; 4 Graduate School of Health Science, Suzuka University of Medical Science, Suzuka, Mie Japan; 5 Present address: Center for Oral Biology, University of Rochester Medical Center, Rochester, NY USA; 6 Present address: Department of Research, Affiliated Tumor Hospital of Guangxi Medical University, Nanning, Guangxi China

**Keywords:** *RERG*, Nasopharyngeal carcinoma, Tumor suppressor gene, DNA methylation, Angiogenesis

## Abstract

**Background:**

Nasopharyngeal carcinoma (NPC) is a malignancy of the head and neck that is prevalent in Southeast Asia and southern China. Recent studies in epigenetics suggest that DNA methylation plays a pivotal role in the onset and progression of cancer. Combining the methyl-DNA binding domain capture technique and cDNA microarray analysis, we identified a unique hypermethylated gene, *RERG* (Ras-like estrogen-regulated growth inhibitor), that was down-regulated in NPC tissues. *RERG* is a tumor suppressor gene that was first reported in breast cancer. However, the functions of *RERG* are largely unknown in other tumor types.

**Methods:**

RERG expression was assessed in human subjects (NPC primary tissues and non-cancer tissues) and cell lines (NPC cell lines and an immortalized epithelial cell line NP460). Further, we investigated the methylation rate of *RERG* in both human subject and cell lines. 5-Aza-2’-deoxycytidine (Aza) or combined with trichostatin A (TSA) were treated to three NPC cell lines (HK1, C666-1 and HK1_EBV). In addition, the role of RERG in NPC cells and its underlying mechanisms were explored by overexpression of RERG in NPC cell lines.

**Results:**

*RERG* was significantly down-regulated in NPC cancer nests compared to normal nasopharyngeal epithelium cells. Furthermore, the *RERG* promoter was frequently methylated in NPC tissues and cell lines. The *RERG* methylation rate yielded an area under the curve (AUC) of receiver operating characteristic (ROC) curve was 0.897 (95%CI: 0.818–0.976). The down-regulation of *RERG* was restored in NPC cells treated with Aza and TSA. In addition, ectopic expression of *RERG* in NPC cell lines resulted in a significant suppression of cell proliferation, clonogenicity, migration and invasion. *RERG*-overexpressing cells showed significantly slower growth and less angiogenesis in tumor xenografts in nude mice. *RERG* suppressed the ERK/NF-κB signaling pathway and inhibited tumor growth and angiogenesis with down-regulation of MMPs and IL8 in tumors of nude mouse xenografts.

**Conclusions:**

Our results suggest that *RERG* is frequently silenced by promoter CpG methylation in NPC, and acts as a functional tumor suppressor by suppressing the ERK/NF-κB signaling pathway. These findings support the potential use of RERG as a novel molecular target in NPC therapy.

**Electronic supplementary material:**

The online version of this article (doi:10.1186/s13046-017-0554-9) contains supplementary material, which is available to authorized users.

## Background

Nasopharyngeal carcinoma (NPC) is a common head and neck malignancy and important health issue in Southeast Asia and southern China although it is rare in Japan and Western countries [1]. It is closely associated with Epstein-Barr virus (EBV) [2] and shows highly invasive and metastatic features [3]. Evidence in recent years suggests that additional genetic and epigenetic abnormalities are necessary to drive the tumorigenic process. Epigenetic changes, which involve DNA methylation and histone modifications, play critical roles in the onset and progression of cancer and other diseases [4, 5]. Hypermethylation of CpG islands is a well-recognized epigenetic event in cancer [6]. Hypermethylated genomic regions frequently mediate the silencing of tumor suppressor genes (TSGs) [6, 7]. Recent studies indicate that epigenetic processes occur very early in cancer development and may contribute to cancer initiation [8]. Epigenetic inactivation of potential TSGs by aberrant promoter DNA methylation has been increasingly recognized to play a key role in the tumorigenesis of NPC by altering gene functions during cell proliferation, apoptosis, and differentiation [2, 7, 9]. The discovery of epigenetic changes may prove to be extremely useful for early detection and prevention of cancer, including NPC [10, 11].

Ras-like estrogen-regulated growth inhibitor (*RERG*) was initially identified as a candidate tumor suppressor gene, and is regulated by estrogen in breast tumors [12, 13]. *RERG* is located on chromosome 12p12 and encodes a small GTP-binding and hydrolyzing protein (GTPase) of the Ras superfamily. *RERG* is widely expressed in multiple normal tissues, while *RERG* expression is lost in breast, kidney, ovary, and colon tumor tissues [12, 14]. It has been reported that *RERG* is a prognostic marker in breast cancer, and its expression has correlated inversely with proliferation, patient survival, and the development of distant metastases [15]. Significantly hypermethylated *RERG* was also observed in colorectal adenocarcinoma [16, 17], and breast cancer [18]. These findings suggest that *RERG* acts as a putative TSG, but the functions of *RERG* in NPC have yet to be identified.

Recent studies indicated that multiple signaling pathways, including mitogen-activated protein kinases (MAPKs), NF-κB, Wnt/β-catenin, EGFR signaling are involved in several human cancers [19–22]. The ERK and NF-κB signaling pathway are known to promote tumor progression and angiogenesis [23–25]. It has been reported that *RERG* was involved in Ras/MEK/ERK signaling pathway in breast cancer [12, 18]. However, the relationship between *RERG* and NF-κB in NPC is still unclear.

Our genome-wide analyses using methyl-capture sequencing and cDNA microarrays have indicated that *RERG* is a candidate TSG in NPC. In the present study, we investigated the epigenetic regulation and potential tumor suppressor function of *RERG* in NPC. *RERG* was found to be epigenetically inactivated in NPC, and ectopic expression of *RERG* reduced cell proliferation, clonogenicity, migration and invasion. In addition, *RERG* suppressed angiogenesis, functioning as a repressor of ERK/NF-κB activation and inhibiting the expression of matrix metalloproteinases (MMPs), interleukin 8 (IL8) and interleukin 6 (IL6) in NPC cells. Overall our data demonstrate that *RERG* acted as a functional TSG by deactivating ERK/NF-κB signaling effectors, and *RERG* is frequently silenced in NPC.

## Methods

### NPC primary tumor biopsies and non-cancerous nasopharyngeal epithelia (NNE)

This study was performed with ethical review committee approval (2009–07–07) from the First Affiliated Hospital of Guangxi Medical University, China, and ethical approval (No. 1116) from Mie University, Japan. As shown in Table 1, primary NPC tumor biopsies were obtained from 52 newly diagnosed and untreated cases (39 males and 13 females, 44.5 ± 13.9 y.o.). The diagnoses were made by experienced pathologists according to the World Health Organization (WHO) classification, and revealed that 98.1% of cases were non-keratinizing carcinoma. A total of 17 NNE samples obtained by tonsillectomy were used as controls (11 males and 6 females, 40.1 ± 14.9 y.o.). Biopsy samples were stored in a freezer at –80 °C. Biopsies of 13 untreated primary NPC tumors (7 males and 6 females, 39.5 ± 7.9 y.o.) and 9 from NNE subjects (4 males and 5 females, 52.2 ± 13.4 y.o.) were obtained for immunohistochemistry (IHC) staining, and all NPC cases were non-keratinizing carcinoma.

**Table 1:** Clinicopathological features of patients

Clinicopathological features	NPC (*n* = 52)	NNE (*n* = 17)
Age (year) mean ± SD	44.5 ± 13.9	40.1 ± 14.9
Male/Female No. (%)	39/13 (75.0/25.0)	11/6 (64.7/35.3)
Histological subtype^a^ No. (%)		
Keratinizing squamous cell carcinoma	1 (1.9)	
Non-keratinizing carcinoma	51(98.1)	
TNM classification^b^ No. ^c^ (%)		
Tumor size No. (%)		
T1	10 (23.3)	
T2	13 (30.2)	
T3	14 (32.6)	
T4	6 (13.9)	
Lymph node metastasis No. (%)		
N0	12 (27.9)	
N1	12 (27.9)	
N2	7 (16.2)	
N3	12 (27.9)	
Metastasis No. (%)		
M0	43 (100)	
M1	0 (0)	
Cancer stage^b^ No. ^c^ (%)		
I- II	13 (30.2)	
III- IV	30 (69.8)	

^a^according to the WHO histological classification of tumors of the nasopharynx

^b^according to the International Union Against Cancer (UICC)

^c^missing of 9 patient data because of no follow-up

### Cell lines

A series of NPC cell lines (HK1, C666-1, HK1_EBV) and the immortalized epithelial cell line NP460 were a kind gift from Professor Sai-Wah Tsao (Hong Kong University) [2, 26–28]. Cells were maintained at 37 °C in a 5% CO_2_ incubator. NPC cells were cultured in RPMI1640 medium (Gibco, Grand Island, NY, USA) supplemented with 10% fetal bovine serum (FBS, Gibco), 100 U/mL penicillin, and 0.1 mg/mL streptomycin. Cells from the immortalized nasopharyngeal epithelial cell line NP460 were maintained in a 1:1 ratio of Defined Keratinocyte-SFM (Gibco) supplemented with growth factors and Epilife medium supplemented with Epilife defined growth supplement (Gibco), 100 U/mL penicillin, and 100 μg/mL streptomycin.

### RNA extraction and quantitative RT-PCR

Total RNA from the biopsy samples and cell lines was isolated with TRIzol reagent (Invitrogen, Carlsbad, CA, USA). Reverse transcription was performed using the miScript II RT kit (Qiagen, Hilden, Germany). Quantitative RT-PCR (qRT-PCR) was performed using the miScript SYBR Green PCR kit (Qiagen) and QuantiTect Primer Assay (Qiagen) by using StepOnePlus Real-Time PCR Systems (Applied Biosystems, Waltham, MA, USA). mRNA expression levels were normalized against the corresponding levels of *GAPDH*. Relative expression levels of NPC relative to NNE were calculated using the ∆∆Ct method [29].

### qPCR to quantify DNA methylation rate

Genomic DNA was extracted from cell lines and biopsy tissues using a QIAamp DNA mini kit (Qiagen). To quantify gene promoter methylation, EpiTect Methyl II PCR Assay (SA Bioscience, Qiagen, Hilden, Germany) was used according to the manufacturer’s instructions. In briefly, genomic DNA was exposed to digestion performed by using EpiTect Methyl II DNA Restriction Kit (SA Bioscience, Qiagen). Four reaction digestions were labelled as: no-enzyme (*Mo*), methylation sensitive enzyme (*Ms*), methylation dependent enzyme (*Md*) and methylation sensitive and dependent enzymes (*Msd*). The reaction digestions were incubated at 37 °C overnight. After incubation, the reactions were stopped by heating-inactivating the enzymes at 65 °C for 20 min. Methyl qPCR was carried out for each of the four digestions (*Mo*, *Ms*, *Md* and *Msd*) using the RT^2^ SYBR Green ROX qPCR Mastermix (SA Bioscience, Qiagen) and EpiTect Methyl II PCR Primer Assay (SA Bioscience, Qiagen).

### DNA bisulfite treatment and promoter methylation analysis

One microgram of genomic DNA was modified by sodium bisulfite by the EpiTect Bisulfit kit (Qiagen). Bisulfite genomic sequencing (BGS) were performed as previously described [30]. BGS was carried out for 40 cycles using Platinum Taq DNA Polymerase High Fidelity (Invitrogen) with BGS primers (*RERG*-BGS-F: GGAGTTTGGAGGTTTGGAAAT and *RERG*-BGS-R: CAAAAACAAATACCAATAACCC). Amplified BGS products were subcloned and transformed into JM109 competent *Escherichia coli* cells (Promega, Madison, WI, USA). At least 5 clones for each sample were randomly chosen for sequencing.

### 5-Aza-2’-deoxycytidine (Aza) and trichostatin A (TSA) treatment

NPC cells (1 × 10^5^) were seeded into 100-mm dishes and allowed to grow overnight. The culture medium was then replaced with fresh medium containing Aza at a final concentration of 10 μmol/L (Sigma-Aldrich, St. Louis, MO, USA). Cells were allowed to grow for 72 h with the Aza-containing medium changed every 24 h, and some were further treated with the histone deacetylase (HDAC) inhibitor TSA (Sigma-Aldrich) for an additional 24 h. Cells were then harvested for RNA and DNA extraction.

### Vector construction and transfection

The full-length open reading frame (ORF) of *RERG* (NM_032918) was cloned into pCMV6-Entry (Origene, Rockville, MD, USA) with SgfI and MluI to generate pCMV6-*RERG*, which carries a flag tag in the N-terminal of *RERG*. Amplified ORF products with ORF primers (*RERG*-CDS-F: GAGGCGATCGCCATGGCTAAAAGTGCGGAG and *RERG*-CDS-R: GCGACGCGTACTACTGATTTTGGTGAGCAT) were subcloned and transformed into JM109 competent *Escherichia coli* cells (Promega, Madison, WI, USA). Construction of recombinant pCMV6-*RERG* was validated by sequencing.

Three NPC cell lines (HK1, C666-1 and HK1_EBV), which showed inactivation of *RERG*, was transfected with the pCMV6- *RERG* plasmid or the empty-vector pCMV6-Entry plasmid using Fugene HD (Promega). After 48 h, cells were harvested for further experiments and selected with G418 (400 μg/mL; Millipore, Darmstadt, Germany) for 2 weeks to obtain stably transfected cell lines. Transfected cell lines were verified by qRT-PCR and western blotting. The stable cells were maintained in medium containing 200 μg/mL G418.

### Cell proliferation assay

The effect of *RERG* on NPC cell proliferation was measured using an MTT assay or a CCK8 assay. NPC cells transfected with empty vector or *RERG* vector were seeded in 96-well plates at a proportionate density and incubated at 37 °C. Subsequently, cell proliferation was assessed every 24 h for the indicated duration. Briefly, 10 μL of MTT (3-(4,5-dimethylthiazol-2-yl)-2,5-diphenyl tetrazolium bromide, 5.0 mg/mL; Sigma-Aldrich) or CCK8 (cell counting kit-8, Dojindo Laboratories, Kumamoto, Japan) was added to each well, and the plates were incubated for 4 h at 37 °C. Optical density (OD) was determined at 570 nm (MTT assay) or 450 nm (CCK8 assay) on a Bio-Rad model 680 microplate reader (Bio-Rad Laboratories, Hercules, CA, USA). OD values reflect the relative number of viable cells.

### Colony formation assay

HK1 cells stably expressing *RERG* or empty vector and HK1_EBV cells transfected with *RERG* or empty vector after 48 h were counted and plated at 500 cells/dish into 60-mm dishes. Then cells were incubated at 37 °C and at an atmosphere of 5% CO_2_ for 2 weeks. Once the cells formed visible colonies, the colonies were washed twice with phosphate-buffered saline and fixed with 70% ethanol for 20 min. The cells were appropriately stained with Giemsa’s solution (Merck, Darmstadt, Germany) and allowed to air dry at room temperature. The experiments were triplicated and the numbers of colonies were microscopically counted.

### In vitro cell migration and invasion assay

Cell migration assays were performed using the CytoSelect Cell Migration Assay kit (membrane filter pore size, 8 μm; Cell BioLabs, San Diego, CA, USA). Cell invasion assays were performed using the CytoSelect 24-Well Cell Invasion Assay (basement membrane, membrane filter pore size, 8 μm; Cell BioLabs). HK1 cells stably expressing *RERG* or empty vector were suspended (0.15 × 10^6^ transfected cells/well) in serum-free medium and placed in the upper chamber, and media containing 10% FBS was placed in the lower well of the migration or invasion plate. After incubation for 24 h and removal of non-migratory cells or non-invasive cells, cells that migrated or passed through the filter were stained, photographed, and counted under a microscope (magnification, ×100 and × 200) for each filter in 3 areas.

### In vivo tumorigenicity assay

Male BALB/c athymic nu/nu mice (5-week-old) (weight range: 17–19 g; Japan SLC, Hamamatsu, Japan) were used to examine tumorigenicity in vivo. The animal experiments were performed according to the Mie University guidelines for laboratory animals (approval No. 26–19). Nude mice were housed in ventilated caging conditions under a 12-h dark/light cycle at constant humidity and temperature. Animals were permitted free access to sterile water and standard laboratory chow. HK1 cells (2 × 10^6^ cells/mouse) stably transfected with *RERG* were injected subcutaneously into the left flank near the forelimb or an equal number of empty vector stable transfected HK1 cells were injected into the right flank. Tumor growth was measured every 3 days for 3 weeks. Tumor volume (mm^3^) was calculated with a caliper (model 530–312; range 0–150 mm; Mitutoyo, Kawasaki, Japan) and evaluated using the formula: V = L × W × H × π/6, where L, W, and H represent tumor diameter in 3 mutually perpendicular planes. Mice were sacrificed by cervical dislocation at the third week after subcutaneous injection. Tumor grafts were then excised and weighed. The tumors were kept in 4% formamide and RNAlater solution for further analysis of IHC and mRNA expression levels, respectively.

### Western blot analysis

Western blot was done according to the standard protocol as described previously [31]. Cultured cells were harvested at 70-80% confluence and lysed using RIPA buffer (Cell Signaling Technology Inc., Dancers, MA, USA) supplemented PMSF (Phenylmethylsulfonyl fluoride, NACALAI TESQUE, INC., Kyoto, Japan). The list of antibodies used in this study is summarized in Additional file 1: Table S1. Signals were detected by LAS4000mini (Fujifilm, Tokyo, Japan), and band intensities of western blots were quantitatively measured integrated grayscale densities in windows of identical size incorporating for each band using ImageJ software (ver. 1.48).

### IHC staining, immunocytochemistry (ICC) staining, and double immunofluorescence staining

For IHC and ICC analysis, standard immunoperoxidase methods were performed as previously described [29]. The list of antibodies used in this study were summarized in Table S1. IHC grading based on intensity and frequency of staining was performed by 2 independent investigators. Staining intensity was scored as negative (0), weak (+1), moderate (+2), or strong (+3). The frequency of positive cells in specific areas was scored as negative (0), less than 25% (+1), 25–50% (+2), 51–75% (+3), or more than 75% (+4), as previously published [32]. The number of cells staining positively for ICC was analyzed using ImageJ software (ver. 1.48).

For immunofluorescence analysis, after deparaffinization and rehydration, antigen was retrieved in 5% urea buffer by microwave heating for 5 min. Sections of 3-μm thickness were incubated overnight at room temperature with the following antibodies: rabbit polyclonal anti-α-SMA-(1:200, Abcam, Cambridge, UK) and mouse monoclonal anti-CD34 (1:200, Monosan, Uden, Netherlands). The sections were next incubated with the following fluorescent secondary antibodies at 1:400 each for 2 h at room temperature (Molecular Probes, Eugene, OR, USA: Alexa 488-labeled goat anti-rabbit IgG antibody; Alexa 594-labeled goat anti-mouse IgG antibody). Finally, the nuclei were stained with 4’,6-diamidino-2-phenylindole (DAPI) and the sections were examined with a fluorescence microscope (BX53, Olympus, Tokyo, Japan).

For digital microscopy assessment of microvessel density, the intensity and area of endothelial or periendothelial cell staining were quantitatively measured using ImageJ software (ver. 1.48). The proportion of blood vessels exhibiting endothelial (CD34-positive) or periendothelial (α-SMA -positive) cells was defined as the area fraction (%) at a magnification of × 200.

### Statistical analysis

All statistical analyses were performed with SPSS v16.0 (SPSS, Chicago, IL, USA). The unpaired Student’s t-test was used to compare the average of 2 groups, and the paired Student’s t-test was used to compare the size and weight of tumor xenografts in the left and right flanks of nude mice. Statistical differences between IHC scores were determined by the Mann-Whitney U test. Receiver operating characteristic (ROC) curves were generated to confirm the accuracy of diagnosis by *RERG methylation rate*, and sensitivity and specificity were computed. *P* <0.05 was considered as statistically significant.

## Results

### RERG is down-regulated and hypermethylated in NPC primary tumors

We performed the methyl-DNA binding domain capture technique and cDNA microarray analysis in seven NPC patients compared to five NNE patients (unpublished data). In the cDNA microarray, 5 genes of RAS type GTPase family were found to be significantly altered. Among these, 3 genes decreased under half and 2 genes increased more than two-fold compared to the NNE group (Additional file 2: Figure S1A). In the Methylated-DNA capture sequencing data, 8 genes of RAS type GTPase family that have an increased more than two-fold level of CpG island methylation relative to NNE group (Additional file 2: Figure S1B). *RERG, RASL11A* and *RASL11B* were marked with DNA promoter methylation and downregulated in NPC tissues (Additional file 2: Figure S1C). *RERG* gene expression was most significantly decreased in cDNA microarray and highly methylated in NPC, which we chose it for our further study.

**Fig. 1:**
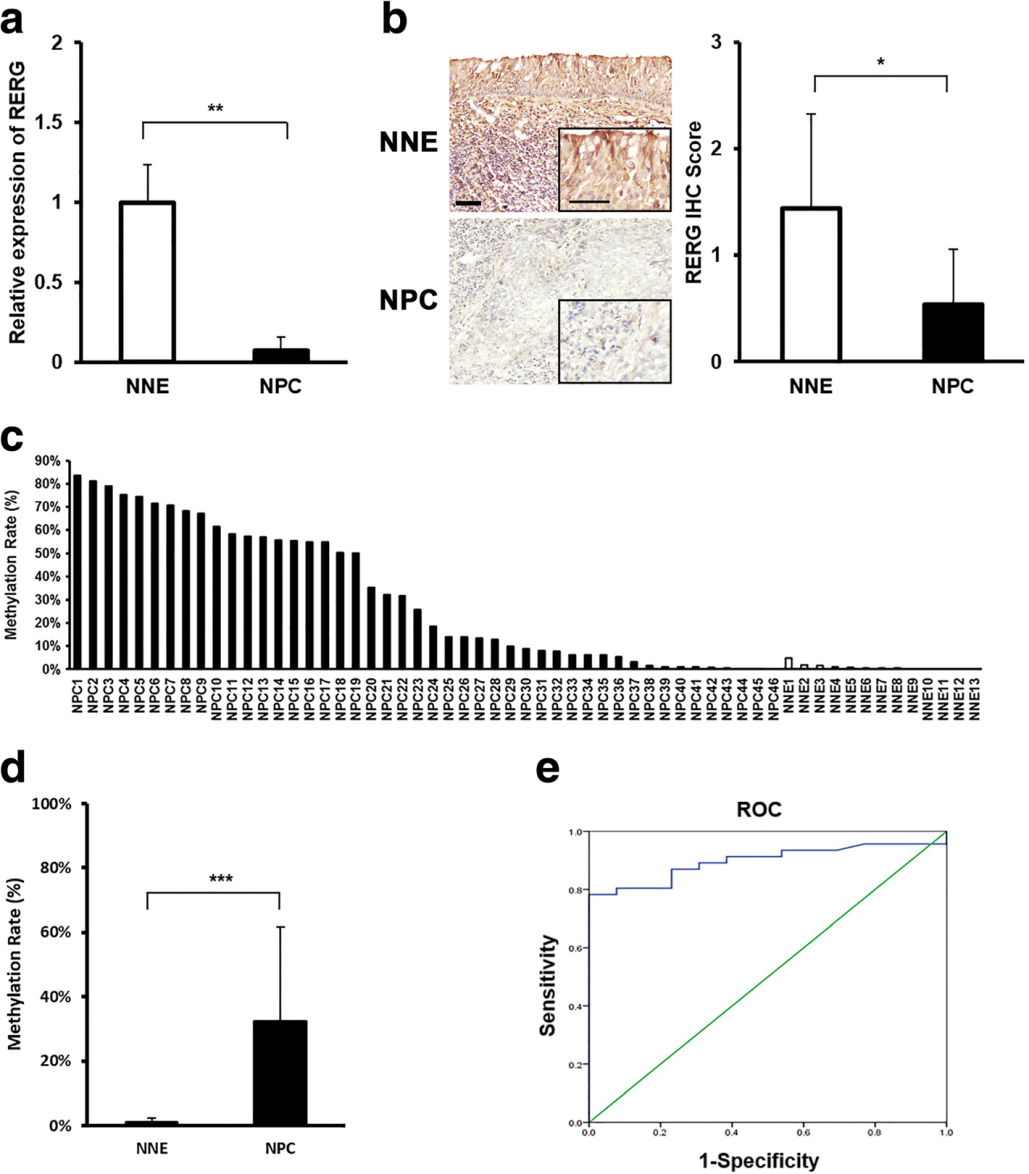
RERG was down-regulated and hypermethylated in NPC primary tumors. **a** Expression levels of *RERG* were measured in NPC primary tumor biopsies (*n* = 16) and NNE tissues (*n* = 13) by qRT-PCR. *GAPDH* was used as an internal control. **: *P* < 0.01 analyzed by Student’s t-test. **b** RERG was down-regulated in NPC clinical samples (*n* = 13) compared to NNE epithelium (*n* = 9) by IHC. *Left*: Representative photographs of IHC analyses of the expression of *RERG*. Original magnification is × 200, inner enlarged magnification is × 400. *Bar* represents 50 μm and 20 μm, respectively. *Right*: *Graphs* represent means ± SD of IHC scores of RERG in tissues of NPC and NNE. *: *P* < 0.05 by Mann-Whitney U test. **c** Each methylation rate (%) of *RERG* in NPC clinical samples and NNE samples by methyl qPCR. **d** Means ± SD of methylation rates of *RERG* in NPC group (n = 46) and NNE group (*n* = 13). ***: *P* < 0.001 by Student’s t-test. **e** ROC analysis of DNA methylation index of *RERG*

We evaluated the expression levels of *RERG* in NPC primary tumors by qRT-PCR and IHC staining. *RERG* expression was significantly lower in NPC primary tumors than NNE tissues (Fig. 1a). IHC staining was then performed to evaluate the expression of RERG proteins in NPC patients and NNE subjects. Moderate expression of RERG (Fig. 1b) was observed in the membrane and cytoplasm of normal nasopharyngeal epithelium cells, while very weak or no expression of RERG was observed in NPC cancer nests. The IHC score of NPC tissues was significantly lower than that of NNE tissues (Fig. 1b). These results suggest that RERG is down-regulated in NPC primary tumors.

A genome database, accessed using the UCSC Genome Browser (https://genome.ucsc.edu/), revealed that the *RERG* promoter contains a CpG island (CGI). As CGI methylation is a well-recognized epigenetic mechanism of TSGs silencing in cancer [33], we sought to determine the quantitation of methylation at the promoter of *RERG*. We performed methyl qPCR in 46 NPC patients and 13 NNE subjects (Fig. 1c). The methylation rate of *RERG* promoter was significantly higher in the NPC group (Average 32.6%) than in the no-cancer group (1.0%) (Fig. 1d, *P* < 0.001).

In addition, ROC curves was conducted to evaluate whether the methylation of *RERG* could be used to be a potential marker for screening NPC. The area under the curve (AUC) of ROC plots for *RERG* methylation rate in the detection of NPC was 0.897 (95%CI: 0.818–0.976) (Fig. 1e). At a meth-index cutoff value of 5.2%, the methylation rate of *RERG* gave 78.3% sensitivity and 100% specificity. The current results indicated that DNA methylation rate of *RERG* holds some promise for NPC screening.

### RERG is silenced in NPC cell lines with DNA hypermethylation and restored by pharmacologic demethylation

**Fig. 2:**
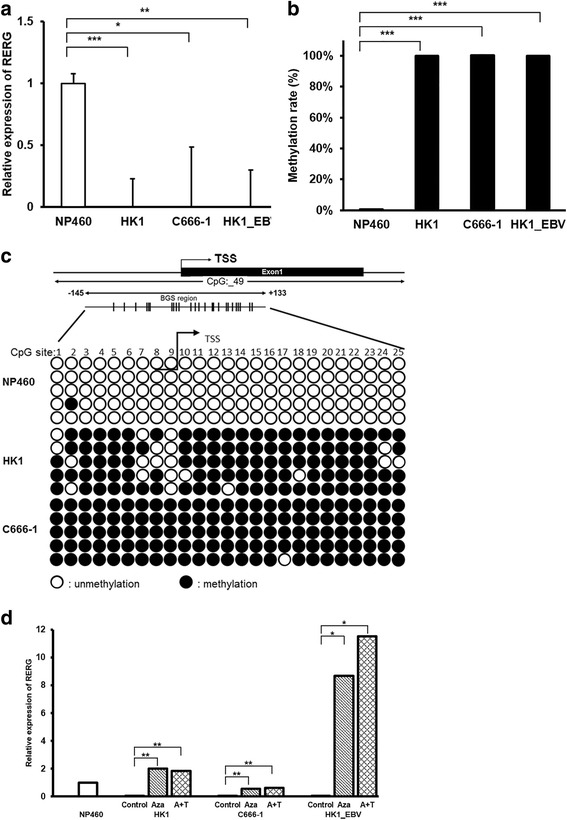
*RERG* was down-regulated and hypermethylated in NPC cell lines, and restored by pharmacologic demethylation. **a** Gene expression was detected in tumor cell lines (HK1, C666-1, HK1_EBV) and an immortalized normal epithelia NP460 cells (*n* = 4) by qRT-PCR. *GAPDH* was used as an internal control. **b** *RERG* was highly methylated in NPC cell lines (HK1, C666-1, HK1_EBV) compared to NP460 (*n* = 3) by methyl qPCR. **c** Bisulfite genomic sequencing of the 25 CpG sites within the *RERG* promoter region in 2 NPC cell lines (HK1 and C666-1) and an immortalized epithelial cell line (NP460). Five clones were randomly selected and sequenced for each sample. Each row represents analysis of an individual promoter allele. *Open circles* indicate unmethylated cytosines, and *filled circles* indicate methylated cytosines. **d** *RERG* expression of NP460 and NPC cell lines (*n* = 3) treated with 5-aza-2’-deoxycytidine (Aza) alone or combined with trichostatin A (TSA) (A + T) were determined by qRT-PCR. *GAPDH* was used as an internal control. *: *P* < 0.05, **: *P* < 0.01, ***: *P* < 0.001 analyzed by Student’s t-test

We also investigated the mRNA expression of *RERG* in cell lines. *RERG* was found to be silenced to a greater extent in NPC cell lines HK1, C666-1 and HK1_EBV compared to an immortalized epithelial cell line (NP460) (Fig. 2a).

We then determined the methylation rate of *RERG* promoter in cell lines. As expected, *RERG* was highly methylated in all 3 NPC cell lines compared to the immortalized normal epithelial cell line, NP460 (Fig. 2b). To further investigate methylation status, we used BGS to determine 25 individual CpG sites from -145 to +133 bp from the transcription start site of the *RERG* promoter. All 25 CpG sites were obviously methylated in NPC cell lines (HK1 and C666-1), but were seldom methylated in the normal cell line, NP460 (Fig. 2c). These results indicate that *RERG* methylation is a common event in NPC.

To investigate whether CpG methylation directly mediates *RERG* down-regulation, 3 NPC cell lines were treated with the DNA methyltransferase inhibitor Aza either alone or in combination with the HDAC inhibitor TSA (A + T). We found that *RERG* expression levels were significantly restored after the demethylation treatment (Fig. 2d), suggesting that promoter methylation might contribute to the silencing of *RERG* in NPC cells. In addition, the mRNA expression levels of *RERG* were up-regulated in demethylated HK1 (2.0 and 1.85 fold with Aza alone and Aza + TSA, respectively) and C666-1 cells (0.57 and 0.62 fold with Aza alone and Aza + TSA, respectively) closed to the immortalized epithelial cell line NP460. *RERG* was highly up-regulated in demethylated HK1_EBV (8.66 and 11.51 fold with Aza alone and Aza + TSA, respectively) than NP460.

### Overexpression of RERG suppresses cell proliferation, clonogenicity, migration, and invasion

**Fig. 3:**
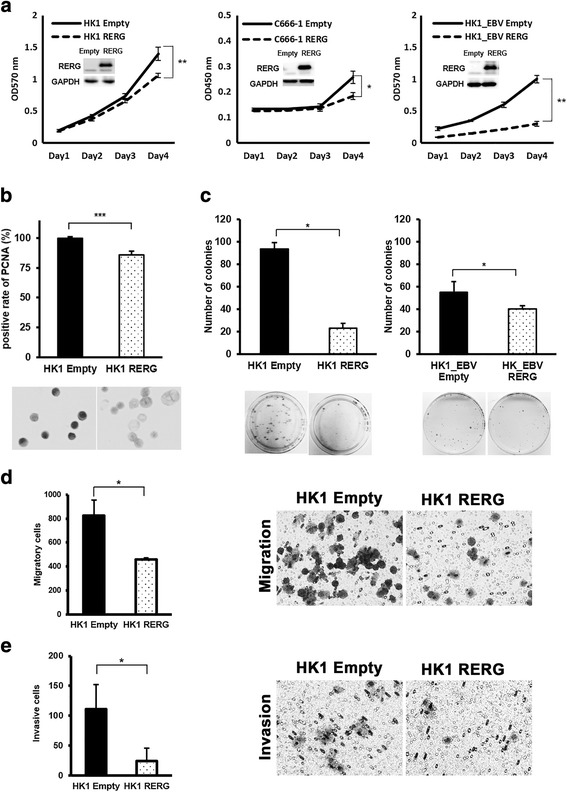
Overexpression of RERG inhibited cell proliferation, colony formation, migration and invasion. **a** Growth curves of *RERG*-transfected and empty-vector-transfected NPC cells (HK1, C666-1, HK1_EBV). Data are means ± SD (n = 5). The insets show protein levels of RERG in each cell line transfected with empty and *RERG* vector. **b** ICC staining of HK1 stable cells against PCNA (magnification, ×200). PCNA-positive percentages in cultured HK1-RERG cells and HK1-empty cells (control) are presented as means ± SD (n = 4). **c** Colony formation assays. Cell colonies were stained with Giemsa. Quantitative analyses of colony formation efficiency show the means ± SD (n = 3) of at least 2 independent experiments. **d** Migration assay. Cells were seeded in a migration chamber and cultured for 24 h. Migratory cells were stained, photographed under a microscope and quantitatively analyzed. **e** Invasion assay. Cells were seeded in an upper chamber and cultured for 24 h. Invasive cells were stained, photographed under a microscope and quantitatively analyzed. Data are shown as means ± SD (n = 3) of at least 2 independent experiments. *: *P* < 0.05, **: *P* < 0.01, ***: *P* < 0.001 by Student’s t-test

To assess the biological roles of RERG in NPC cells, we performed transfection of *RERG*-expression vector in 3 NPC cell lines (HK1, C666-1 and HK1_EBV). We established one stable RERG-expressing HK1 cells. The overexpression of RERG was confirmed by western blot (Fig. 3a, inset). Overexpression of RERG significantly inhibited NPC cell proliferation (Fig. 3a). Moreover, the proliferative effect of RERG was confirmed via ICC staining of PCNA, a marker of cell proliferation in HK1 stable cells. The number of PCNA-positive cells was significantly reduced by 15% in HK1-RERG cells (Fig. 3b). RERG also significantly reduced the colony formation (Fig. 3c), migration efficiency (Fig. 3d) and invasion (Fig. 3e) of NPC cells, when compared with empty-vector cells.

### Overexpression of RERG suppresses MMPs and pro-angiogenic factors via the ERK and NF-κB signaling pathway in NPC cells

**Fig. 4:**
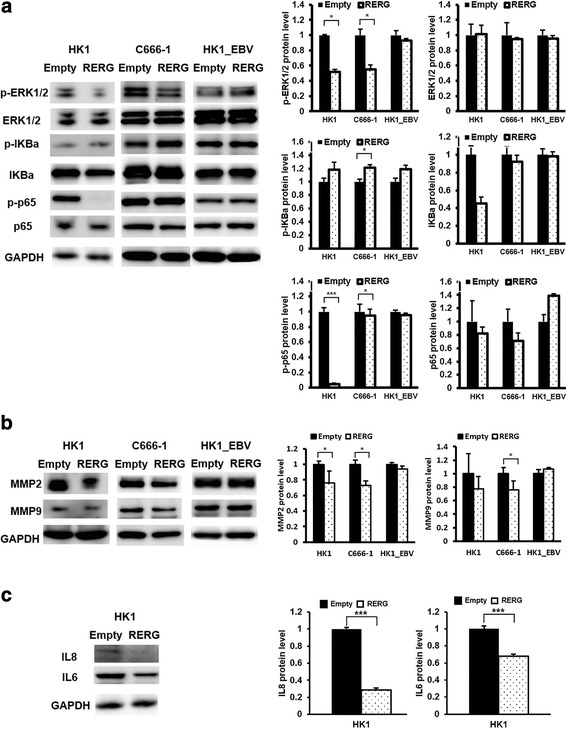
RERG suppressed ERK/NF-κB signaling pathway and expression of MMPs, IL8 and IL6 in NPC cells. Protein levels of (**a**) ERK/NF-κB signaling effectors and (**b**) MMP2 and MMP9 in *RERG*-transfected and empty-vector-transfected NPC cells (HK1, C666-1, HK1_EBV) were determined by western blotting (*n* = 3). **c** IL8 and IL6 in *RERG*-transfected and empty-vector-transfected NPC cells (HK1) were determined by western blotting (*n* = 3). Data are shown as means ± SD. *: *P* < 0.05, **: *P* < 0.01, ***: *P* < 0.001 by Student’s t-test

To explore the mechanism of tumor suppression by RERG, we compared the expression levels of major ERK and NF-κB signaling pathway members, prominent cell migration and invasion molecules (MMP-2 and MMP-9), and several pro-angiogenesis-related factors, such as vascular endothelial growth factor (VEGF), IL8 and IL6 in NPC cells. RERG reduced p-ERK and p-p65, and increased p-IKBα protein levels in NPC cell lines, as typically seen in HK1 stable cells and C666-1 cells (Fig. 4a). Also, MMPs were suppressed by the overexpression of RERG (Fig. 4b). The significant downregulation of IL8 and IL6 were confirmed in HK1 stable cell by western blotting (Fig. 4c). However, RERG showed limited effects on VEGF when compared to empty-vector cells (Fig. S2A). As expected, RERG suppressed ERK and NF-κB signaling pathway and its downstream effectors. It has been reported that both Ras/ERK and NF-κB signaling are significantly associated with cell proliferation, survival, differentiation, and commonly involved in malignant transformation in a variety of human cancers [34]. Thus, these results suggested that RERG overexpression exerted a tumor suppressor role by attenuating the activation of ERK/NF-κB signaling pathway.

### Overexpression of RERG suppresses tumorigenesis and angiogenesis in vivo

**Fig. 5:**
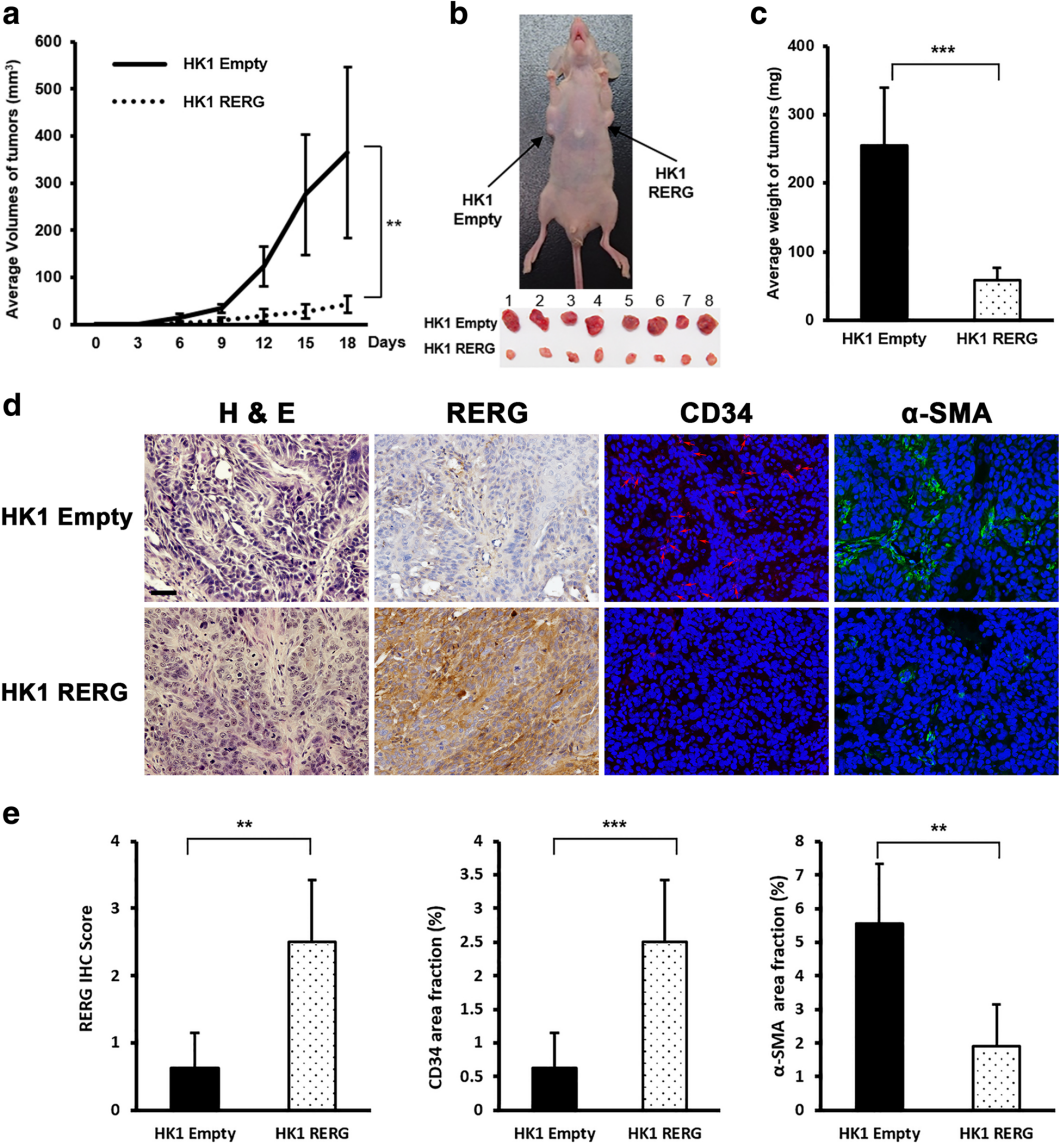
RERG inhibited the tumorigenesis and angiogenesis of NPC in vivo. Eight male BALB/c athymic nu/nu mice injected with 2 × 10^6^ cells. **a** Growth curve of tumors in nude mice. Tumor volume was measured every 3 days after inoculation. **b** Image of nude mouse tumors derived from HK1 cells stably transfected with *RERG* or empty vector. *Arrows* indicate positions and locations of tumors. **c** The average weights of tumors in nude mice. **d** Representative photographs of H&E staining, IHC analyses of the expression of RERG and immunofluorescence analyses of the expression of CD34 (*red*), α-SMA (*green*) in tumors from nude mice. Nuclei were counterstained by DAPI (*blue*) in the merged pictures of immunofluorescence analyses. Original magnification is × 200. *Bar* represents 50 μm. **e** *Left*, IHC scores of RERG in tumors from nude mice. *Middle* and *right*, for immunofluorescence analyses, *graphs* represent average area fraction (%) ± SD of microvessels/field by CD34 and α-SMA area fraction (%) in tumors from nude mice analyzed by Image J. Data are shown as means ± SD. **: *P* < 0.01, ***: *P* < 0.001 by Mann-Whitney U test or Student’s t-test

We further tested whether RERG is able to suppress the growth of NPC cells in vivo. Stable transfected HK1-RERG and HK1-Empty cells formed tumors in nude mice. A 100% tumor formation rate suggested that *RERG* did not affect tumor incidence in vivo. However, the subcutaneous tumor growth curve was significantly less steep for *RERG*-transfected cells than for controls (Fig. 5a). After 19 days, xenografts were removed from mice (Figs. 5b). The mean weight of tumors formed by *RERG*-transfected cells was significantly lower than that of controls (59.2 ± 17.3 mg vs 254.6 ± 84.9 mg; Fig. 5c), indicating that RERG can inhibit NPC tumorigenicity in vivo.

RERG expression was confirmed by IHC (Fig. 5d, e) and qRT-PCR (Additional file 3: Figure S2B). In a histopathological analysis, H&E staining showed that angiogenesis was suppressed in xenografts from the RERG group (Fig. 5d). To study the potential effect of RERG on angiogenesis, the development of microvessels in tumor sections of nude mice was examined by immunofluorescence staining with vascular endothelial cell marker CD34 and periendothelial cell marker α-SMA (Fig. 5d). Area fraction of CD34 and α-SMA was significantly lower in the mouse xenografts within the RERG-overexpressed tumors (Fig. 5e).

### RERG mediates downregulation of MMPs and pro-angiogenic cytokines with the inhibition of NF-κB signaling

**Fig. 6:**
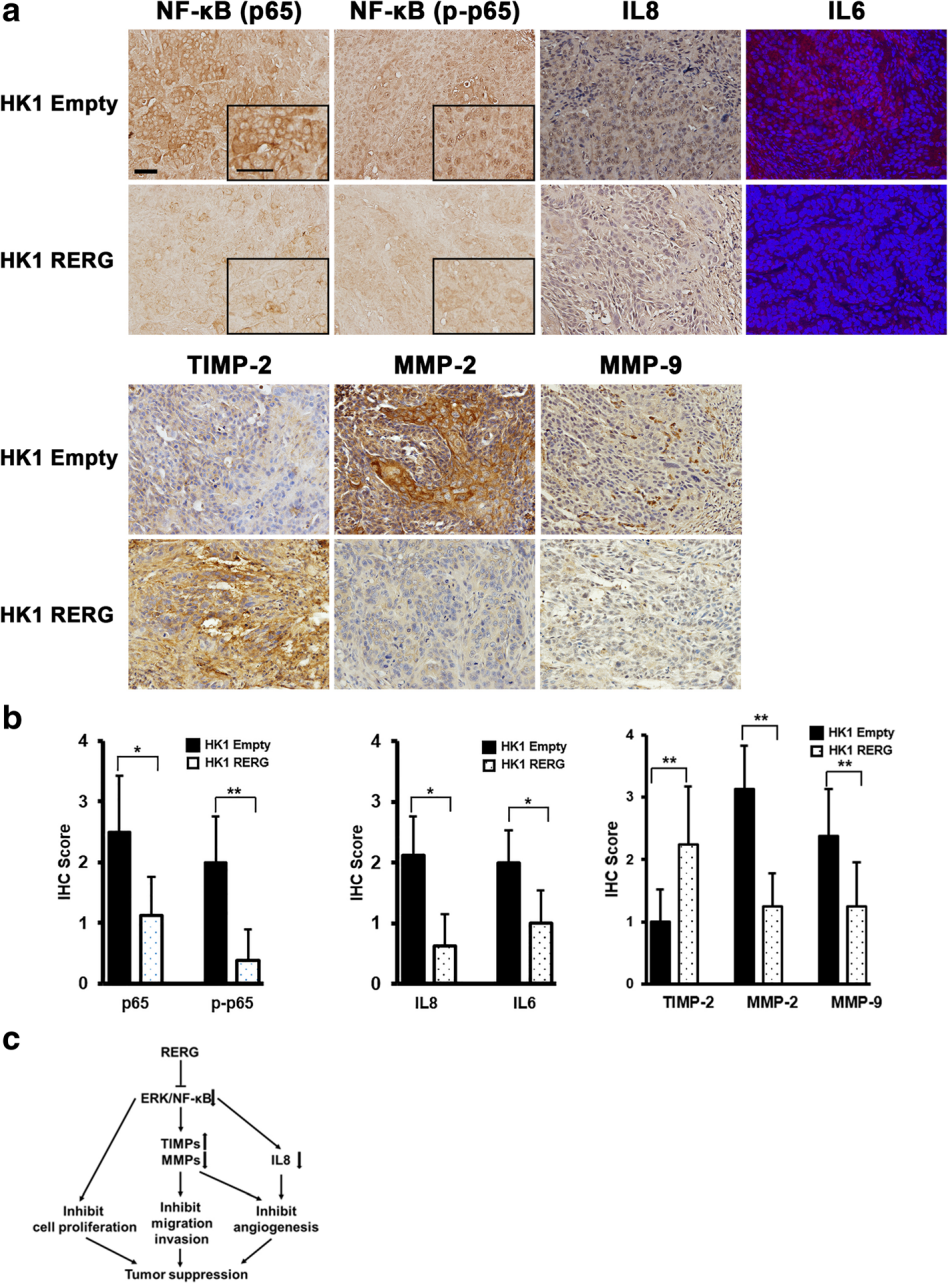
RERG suppressed MMPs and pro-angiogenic factors through inhibition of NF-κB signaling. **a** IHC analyses or immunofluorescence analyses of the expression of NF-κB (p65 and p-p65), IL8, IL6, TIMP-2, MMP-2, and MMP-9 in tumors from nude mice. Nuclei were counterstained by DAPI (*blue*) in the merged pictures of immunofluorescence analyses. Original magnification is × 200 inner enlarged magnification is × 400. *Bar* represents 50 μm and 20 μm, respectively. **b** IHC scores of NF-κB (p65 and p-p65), IL8, IL6, TIMP-2, MMP-2, and MMP-9 in tumors from nude mice. **: *P* < 0.01, ***: *P* < 0.001 by Mann-Whitney U test. **c** Schematic diagram for the molecular basis of *RERG* as a tumor suppressor gene in NPC, based on decreasing cell proliferation and inhibiting migration, invasion and angiogenesis through the ERK/NF-κB signaling pathway

The NF-κB signaling pathway is known to promote tumor progression and angiogenesis [24, 25]. To understand how NF-κB signaling pathway is regulated by RERG in vivo, we focused on the subunit p65 translocation into the cell nuclei. IHC staining indicated that p-p65 localized exclusively in the nuclei of control mouse xenografts and slightly in the RERG-overexpressed mouse xenografts tumors (Fig. 6a). Interestingly, a decrease in total p65 expression was also observed in RERG-overexpressed tumors (Fig. 6a). It is indicated that expression of RERG inhibited p65 nuclear translocation in vivo. We then confirmed pro-angiogenic cytokines (IL8 and IL6), TIMP-2, and MMPs (MMP-2 and MMP-9) in the isolated tumor tissues by IHC (Fig. 6a). Pro-angiogenic cytokines IL8 and IL6 were significantly downregulated in RERG-overexpressed tumors (Fig. 6b). IHC scores (Fig. 6b) indicated that overexpression of RERG significantly decreased the expression of MMPs (MMP-2 and MMP-9), and drastically up-regulated TIMP-2, which is an inhibitor of MMPs, including MMP-2 and MMP-9. Real-time qRT-PCR validated that the mRNA expression of *IL8* and *IL1β* were downregulated by *RERG* in mouse xenografts tumors (Additional file 3: Figure S2B). On the other hand, the expression of TNFα and VEGF showed no significant changes in vivo (Additional file 3: Figure S2B, C). These results suggested that RERG efficiently inhibited the cell proliferation, migration, invasion and angiogenesis of NPC cells by suppressing the cell migratory and pro-angiogenic molecules (MMP-2, MMP-9, IL8, IL6 and IL1β) via the ERK/NF-κB signaling pathway (Fig. 6c), but not VEGF and TNFα.

## Discussion

In this study, we found that *RERG* was frequently silenced in NPC tissues and cell lines. As mRNA expression of *RERG* was pharmacologically reversible, promoter methylation seems to be the major mechanism of inactivating *RERG*, and histone modification may partly co-enhance its demethylation effect. Epigenetic gene silencing is associated with the onset and progression of various cancers [7]. The frequent, predominant epigenetic inactivation of *RERG* in NPC points to the importance of this gene in tumorigenesis. We also found that *RERG* exerts its tumor suppressor functions by inhibiting ERK/NF-κB signaling pathway, resulting in suppression of tumor cell proliferation, clonogenicity, migration, invasion and angiogenesis (Fig. 6c). These results suggest that RERG suppresses cellular growth through signaling pathways in which these molecules participate. Although this and other studies have demonstrated that RERG is down-regulated in multiple cancers, few studies have reported on its function and related mechanisms in tumorigenesis. To the best of our knowledge this study is the first to reveal the underlying antitumor mechanisms of RERG in NPC.

RERG is a member of RAS GTPase superfamily, which had previously been implicated regulating Ras/MEK/ERK signaling pathway activation [12]. The Ras/MEK//ERK pathway, one of mitogen-activated protein kinases (MAPKs), function in a variety of cellular regulation leading to cell growth and development, and is hyperactivated in a variety of human cancers [34], such as gastric adenocarcinoma, hepatocarcinoma, colon cancer [35, 36] and NPC [37]. Phosphorylation of ERK proteins via the Ras/MEK/ERK signaling pathway cascade induces the activation of transcription factors NF-κB, AP-1, and ETS [20]. NF-κB is also a well-known master regulator of host inflammatory responses and NF-κB activation is a common event in human cancers, including NPC [38]. Activation of NF-κB influences the proliferation, survival, invasive, and metastatic properties of cancer cells by regulating the transcription of various important target genes [39]. Our results indicate that overexpression of RERG inhibited ERK/NF-κB in several NPC cell lines, and reduced total p65 and activated p65 both in vitro and in vivo. Several reports indicated that the inhibition of NF-κB activity in NPC cells resulted in the down-regulation of downstream target genes such as MMPs and IL8 [40, 41]. Therefore, it is conceivable that RERG affects the ERK/NF-κB signaling pathway in NPC cells.

The tumor microenvironment, composed of non-cancer cells and their stroma, influences on metastasis as a dynamic process [42] and angiogenesis response to a tumor [43]. Various of pro-tumorigenic factors, including MMPs and cytokines are produced by tumor cells as well as by the tumor stroma. MMPs, a family of zinc metallo-endopeptidases capable of digesting the extracellular matrix and basement membrane, promote tumor invasion and metastasis [44]. Decreased MMP expression and activity can inhibit cancer cell adhesion, migration, invasion and angiogenesis. In this study, we confirmed that ectopic expression of RERG reduced MMP-2 and MMP-9 expression in vitro and in vivo, although some differences were observed between the results of in vitro and in vivo. In mouse xenografts tumors model, the MMPs can be secreted by cancer cells as well as cancer-associated fibroblasts (CAF). The crosstalk between cancer cells and tumor stroma in the tumor microenvironment enhances the production of pro-angiogenic protein, including MMPs. A possible explanation is that overexpression of RERG might suppress the crosstalk between cancer cells and tumor stroma in vivo, but not in vitro. Additionally, IHC showed that TIMP-2, an inhibitor of MMPs, was up-regulated in mouse xenografts. It was reported that TIMPs exert antiangiogenic activity by suppressing MMPs or by directly inhibiting endothelial cell proliferation [45]. An imbalance between MMPs and TIMPs might result in the deposition or degradation of the extracellular matrix [46]. In the present study, therefore, we speculated that the overexpression of RERG mediated the suppression of ERK/NF-κB pathway, reducing the expression of MMP-2 and MMP-9 in NPC. These results might provide a molecular basis for its ability to inhibit cell proliferation, migration and invasion.

In addition, we observed that in a mouse xenograft model, RERG inhibited microvessel development as well as tumor growth. CD34 (an endothelial cell marker) and α-SMA (a pericyte marker) was dramatically decreased in the mouse xenografts within the RERG-expression. Our previous IHC study suggested that α-SMA-positive stromal cells may recruit circulating endothelial progenitor cells into NPC stroma for angiogenesis [32]. As α-SMA is widely accepted as CAF marker, there is a possibility of the activation and the recruitment of the stromal cells, and it may be inhibited by the over-expression of the novel tumor suppressor gene (*RERG*). As overexpression of RERG reduced the expression of α-SMA in mouse xenograft model, it is suggested that RERG might involve in the suppressing the crosstalk between tumor cells and tumor microenvironment.

Angiogenesis is a highly regulated process involving sprouting from pre-existing vessels and maturation into new blood vessels [47]. It can be triggered and modified by various factors, including cytokines, growth factors and their receptors, chemokines, and matrix metalloproteinases [47]. However, there are no studies on the relationship between angiogenesis and RERG. VEGF is one of the most well-known pro-angiogenic factors, stimulating the sprouting and proliferation of endothelia cells [43]. IL8 is a pro-angiogenesis cytokine, which may promote both tumor growth and the formation of new blood vessels [48, 49]. IL6 promotes the migration and invasion in NPC cell lines and mediated through regulation of the expression of MMP-2 and MMP-9 [50]. IL-1 α and β were detectable in the majority of primary NPC biopsies and a fraction of metastatic lesions and its absence in control nasopharyngeal tissues [51]. In this study, RERG decreased IL8, IL6 and IL1β expression and angiogenesis in tumor xenografts in nude mice. In addition, the protease MMPs were well-known pro-angiogenic factors in the angiogenesis pathway [43, 52, 53]. RERG increased the expression of TIMP-2 and inhibited the expression of MMP-2 and MMP-9, providing support for a mechanistic explanation of the inhibition of tumor microvessel growth and size by RERG. MMPs, as well as cytokines, IL8 and IL6 were the most down-regulated pro-angiogenic factors, followed re-expression of RERG in NPC. However, the effect of VEGF and TNFα were not significant, indicating that they may not play important roles in suppressing angiogenesis via RERG regulation. This study provides the first evidence that RERG expression can reduce angiogenesis. In this study, we observed that RERG reduced cellular migration and invasion in NPC in vitro and suppressed tumor growth and angiogenesis in vivo, but the potential ability and the detailed mechanisms of RERG-inducing distant metastasis in vivo requires further study.

## Conclusions

We demonstrated that *RERG* functioned as a TSG through suppression of ERK/NF-κB signaling pathway, and was frequently silenced by promoter CpG methylation in NPC. This study provides insights into the possible mechanisms underlying the role of RERG in NPC carcinogenesis, and suggests that RERG might be employed as a target molecule in cancer therapy.

## Additional files

Additional file 1: Table S1. List of primary antibodies used in the study. (DOCX 16 kb)

Additional file 2: Figure S1. Analysis of RAS type GTPase family genes in nasopharyngeal carcinoma primary tumors and nasopharyngeal epithelial tissues. (A, B) Heat map of RAS type GTPase family in NPC (*n* = 7) and NNE (*n* = 5) tissues. (A) RAS type GTPase family genes expression alerted using cDNA microarray. (B) Hypermethylated genes of RAS type GTPase family by methylated-DNA capture sequencing. (C) Genes of RAS type GTPase family which were significantly downregulated in cDNA microarray and hypermethylated in methylated-DNA capture sequencing. Methods for methyl-capture sequencing and gene expression array were described in Additional file 4: Supplementary methods. (TIF 2684 kb)

Additional file 3: Figure S2. Analysis of VEGF and SASP factors in vitro and in vivo. (A) VEGF in *RERG*-transfected and empty-vector-transfected NPC cells (HK1, C666-1) were determined by western blotting (*n* = 3). (B) mRNA expression of *RERG*, *IL8*, *IL1β* and *TNFα* in xenografts of nude mice was determined by qRT-PCR (*n* = 8). *GAPDH* was used as an internal control. (C) IHC analyses of the expression of VEGF in tumors from nude mice. Original magnification is × 200. Bar represents 50 μm. Data are shown as means ± SD. **: P < 0.01, ***: P < 0.001 by Student’s t-test or Mann-Whitney U test. (JPG 1577 kb)

Additional file 4: Supplementary methods. (DOCX 14 kb)

## Abbreviations

AUC: Area under the curve
Aza: 5-Aza-2’-deoxycytidine
BGS: Bisulfite genomic sequencing
CCK8: Cell counting kit-8
DAPI: 4’,6-diamidino-2-phenylindole
EBV: Epstein-Barr virus
HDAC: Histone deacetylase
IHC: Immunohistochemistry
IL6: Interleukin-6
IL8: Interleukin-8
MMPs: Matrix metalloproteinases
MTT: 3-(4,5-dimethylthiazol-2-yl)-2,5-diphenyl tetrazolium bromide
NNE: Non-cancerous nasopharyngeal epithelia
NPC: Nasopharyngeal carcinoma
PCNA: Proliferating cell nuclear antigen
*RERG*: Ras- like estrogen-regulated growth inhibitor
ROC: Receiver operating characteristic
TIMPs: Tissue inhibitors of metalloproteinases
TNFα: Tumor necrosis factor-α
TSA: Trichostatin A
TSG: Tumor suppressor gene
VEGF: Vascular endothelial growth factor
