# Erratum to: *RERG* suppresses cell proliferation, migration and angiogenesis through ERK/NF-κB signaling pathway in nasopharyngeal carcinoma

**DOI:** 10.1186/s13046-017-0565-6

**Published:** 2017-07-13

**Authors:** Weilin Zhao, Ning Ma, Shumin Wang, Yingxi Mo, Zhe Zhang, Guangwu Huang, Kaoru Midorikawa, Yusuke Hiraku, Shinji Oikawa, Mariko Murata, Kazuhiko Takeuchi

**Affiliations:** 1 Department of Environmental and Molecular Medicine, Mie University Graduate School of Medicine, 2-174, Edobashi, Tsu, Mie 514-8507 Japan; 2 Department of Otorhinolaryngology - Head and Neck Surgery, Mie University Graduate School of Medicine, 2-174, Edobashi, Tsu, Mie 514-8507 Japan; 3 Department of Otolaryngology Head and Neck Surgery, First Affiliated Hospital of Guangxi Medical University, Nanning, Guangxi China; 4 Graduate School of Health Science, Suzuka University of Medical Science, Suzuka, Mie Japan; 5 Present address: Center for Oral Biology, University of Rochester Medical Center, Rochester, NY USA; 6 Present address: Department of Research, Affiliated Tumor Hospital of Guangxi Medical University, Nanning, Guangxi China

## Erratum

Upon publication of the original article [1], it was noticed that Fig. 5 contained an error. The Fig. 5e (middle) for CD34 area fraction (%) data was erroneously included during the drafting of the manuscript. This has now been acknowledged and corrected in this erratum. The correct Fig. 5 is shown below.

**Fig. 5:**
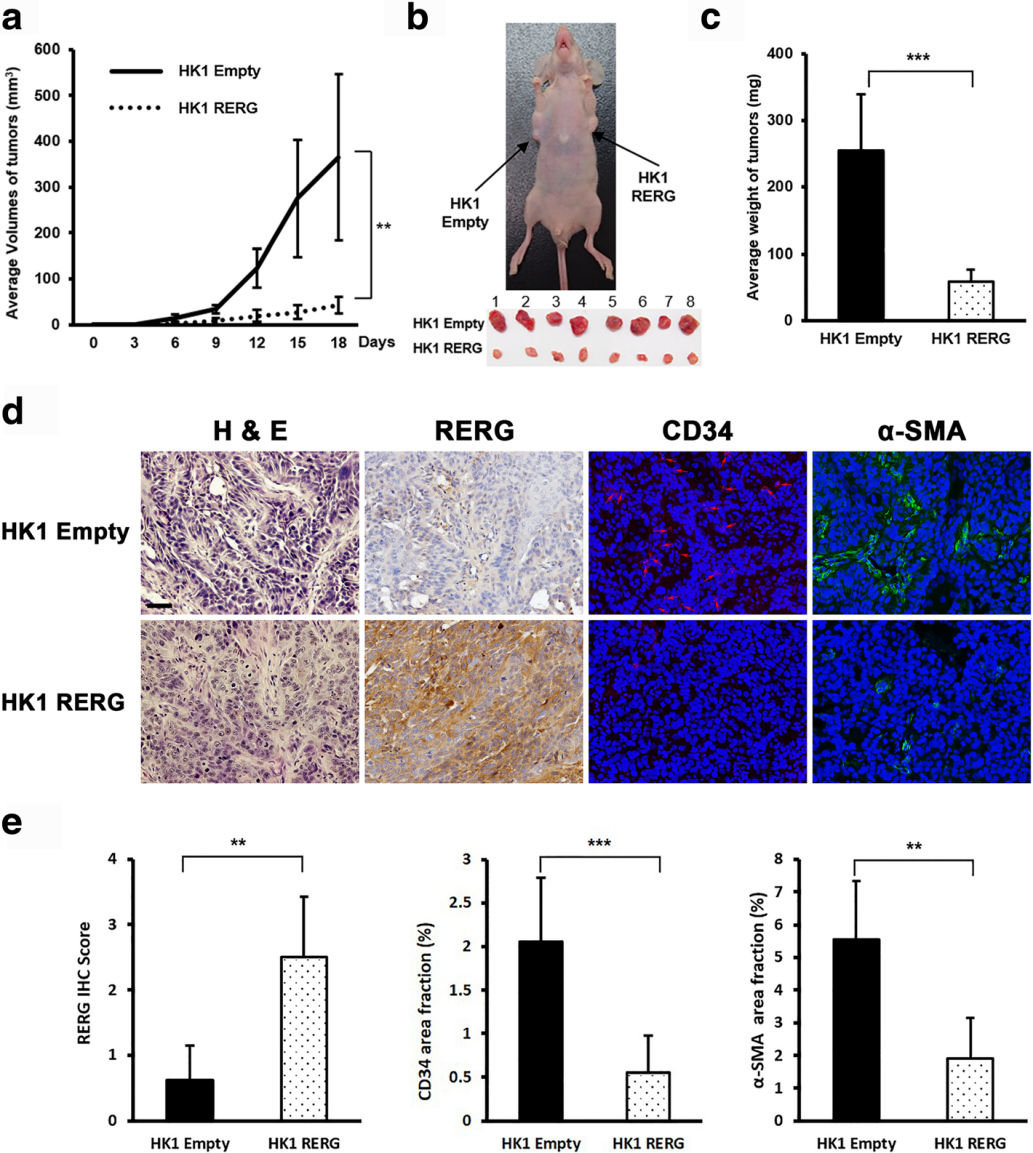
RERG inhibited the tumorigenesis and angiogenesis of NPC in vivo. Eight male BALB/c athymic nu/nu mice injected with 2 × 10^6^ cells. **a** Growth curve of tumors in nude mice. Tumor volume was measured every 3 days after inoculation. **b** Image of nude mouse tumors derived from HK1 cells stably transfected with *RERG* or empty vector. *Arrows* indicate positions and locations of tumors. **c** The average weights of tumors in nude mice. **d** Representative photographs of H&E staining, IHC analyses of the expression of RERG and immunofluorescence analyses of the expression of CD34 (*red*), α-SMA (*green*) in tumors from nude mice. Nuclei were counterstained by DAPI (*blue*) in the merged pictures of immunofluorescence analyses. Original magnification is × 200. *Bar* represents 50 μm. **e** *Left*, IHC scores of RERG in tumors from nude mice. *Middle* and *right*, for immunofluorescence analyses, *graphs* represent average area fraction (%) ± SD of microvessels/field by CD34 and α-SMA area fraction (%) in tumors from nude mice analyzed by Image J. Data are shown as means ± SD. **: *P* < 0.01, ***: *P* < 0.001 by Mann-Whitney U test or Student’s t-test
