# Supplementary material for: RERG suppresses cell proliferation, migration and angiogenesis through ERK/NF-κB signaling pathway in nasopharyngeal carcinoma
Source: J Exp Clin Cancer Res. 2017 Jun 28;36:88. doi: 10.1186/s13046-017-0554-9 (PMC5490152; doi:10.1186/s13046-017-0554-9)
Supplement: Supplementary file 1 — List of primary antibodies used in the study. (DOCX 16 kb) [file 13046_2017_554_MOESM1_ESM.docx]

Table S1. List of primary antibodies used in the study.

| Antibody name | Source | Catalog No. | Host | Dilution | Size (kDa) | Note |
| --- | --- | --- | --- | --- | --- | --- |
| RERG | Proteintech | 10687-1-AP | Rabbit | 1:2000 | 23 | WB |
| MMP-2 | Cell Signaling Technology Inc. | 4022 | Rabbit | 1:1000 | 72 | WB |
| MMP-9 | Santa Cruz Biotechnology Inc. | sc-6840 | Goat | 1:200 | 92 | WB/IHC |
| p44/42 MAPK (ERK1/2) | Cell Signaling Technology Inc. | 4695 | Rabbit | 1:1000 | 42, 44 | WB |
| Phospho-p44/42 MAPK (ERK1/2)  (Thr202/Tyr204) | Cell Signaling Technology Inc. | 4370 | Rabbit | 1:1000 | 44, 42 | WB |
| p65 | Cell Signaling Technology Inc. | 8242 | Rabbit | 1:1000 | 65 | WB/IHC |
| Phospho-p65 (Ser536) | Cell Signaling Technology Inc. | 3033 | Rabbit | 1:1000 | 65 | WB/IHC |
| IkBα | Cell Signaling Technology Inc. | 4814 | Mouse | 1:1000 | 39 | WB |
| Phospho-IkBα(Ser32/36) | Cell Signaling Technology Inc. | 9246 | Mouse | 1:1000 | 40 | WB |
| GAPDH | Abcam | ab9485 | Rabbit | 1:2500 | 40 | WB |
| RERG | Sigma-Aldrich | HPA041387 | Rabbit | 1:200 |  | IHC |
| TIMP-2 | Santa Cruz Biotechnology Inc. | sc-5539 | Rabbit | 1:200 |  | IHC |
| MMP-2 | Kyowa Pharma Chemical Inc. | F-73 | Mouse | 1:200 |  | IHC |
| PCNA | Novocastra Leica | NCL-L-PCNA | Mouse | 1:100 |  | ICC/IHC |
| p65 | Santa Cruz Biotechnology Inc. | sc-8008 | Mouse | 1:200 |  | IHC |
| CD34 | Monosan | MON-RTU1042 | Mouse | 1:200 |  | IHC |
| 𝛼-SMA | Abcam | ab5694 | Rabbit | 1:200 |  | IHC |
| VEGF | Abcam | ab46154 | Rabbit | 1:1000 | 43 | WB/IHC |
| IL8 | Abcam | ab7747 | Rabbit | 1:100 | 11.1 | WB/IHC |
| IL6 | Abcam | ab6672 | Rabbit | 1:1000 | 25, 50 | WB/IF |
