# Supplementary figures and images for: RERG suppresses cell proliferation, migration and angiogenesis through ERK/NF-κB signaling pathway in nasopharyngeal carcinoma
Source: J Exp Clin Cancer Res. 2017 Jun 28;36:88. doi: 10.1186/s13046-017-0554-9 (PMC5490152; doi:10.1186/s13046-017-0554-9)

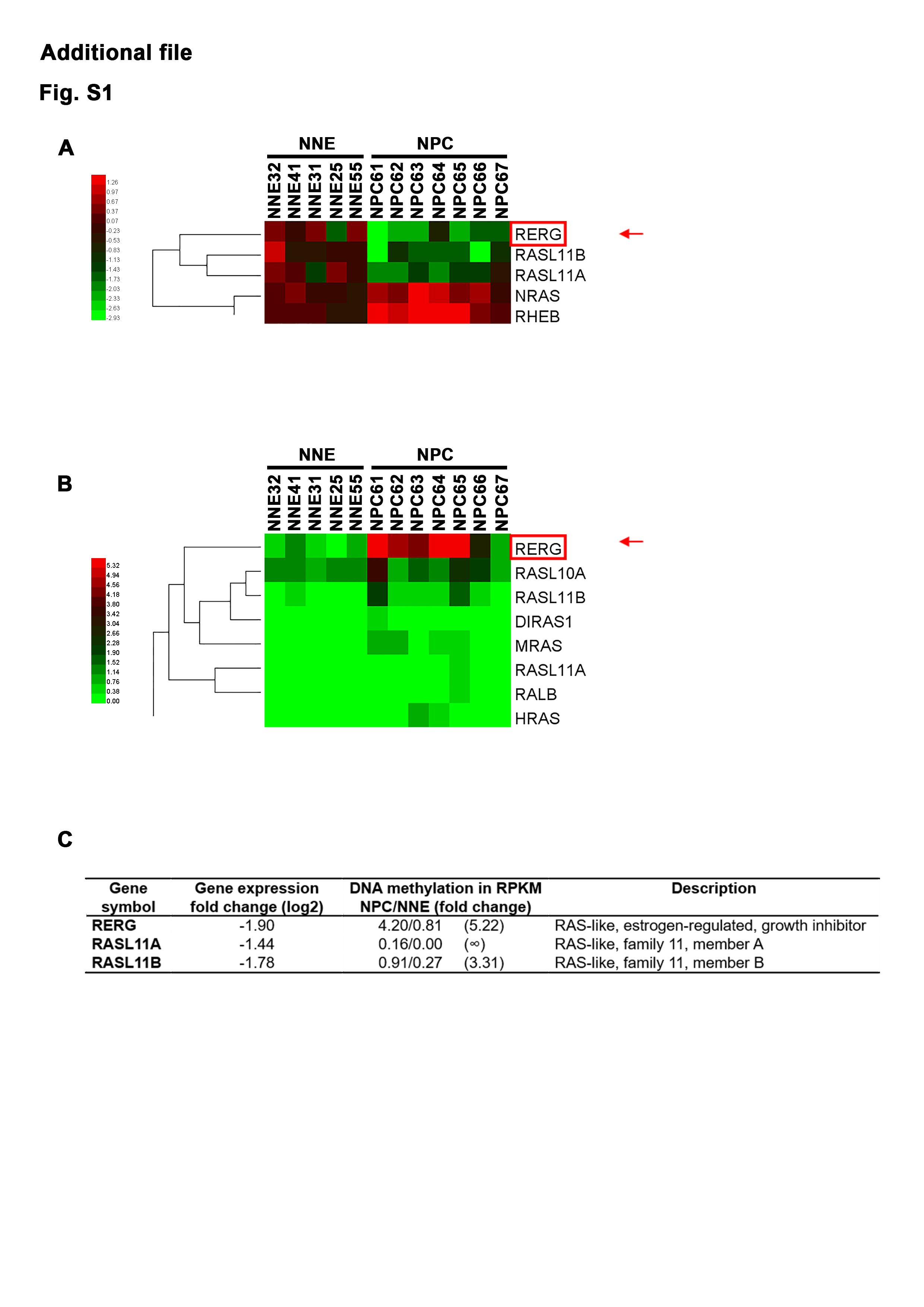

Supplement: Supplementary file 2 — Analysis of RAS type GTPase family genes in nasopharyngeal carcinoma primary tumors and nasopharyngeal epithelial tissues. (A, B) Heat map of RAS type GTPase family in NPC (n = 7) and NNE (n = 5) tissues. (A) RAS type GTPase family genes expression alerted using cDNA microarray. (B) Hypermethylated genes of RAS type GTPase family by methylated-DNA capture sequencing. (C) Genes of RAS type GTPase family which were significantly downregulated in cDNA microarray and hypermethylated in methylated-DNA capture sequencing. Methods for methyl-capture sequencing and gene expression array were described in Additional file 4: Supplementary methods. (TIF 2684 kb) [file 13046_2017_554_MOESM2_ESM.tif]

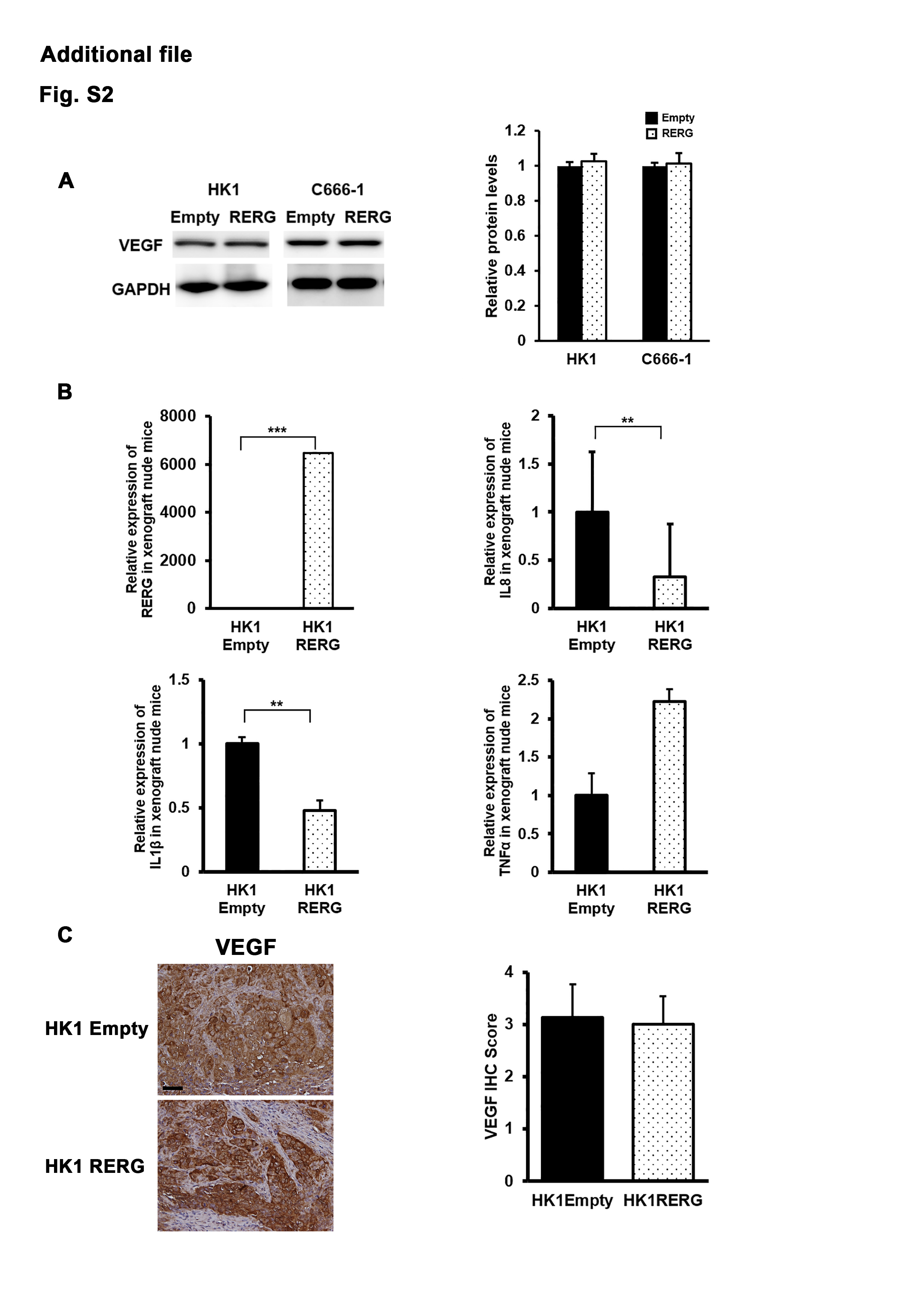

Supplement: Supplementary file 3 — Analysis of VEGF and SASP factors in vitro and in vivo. (A) VEGF in RERG-transfected and empty-vector-transfected NPC cells (HK1, C666-1) were determined by western blotting (n = 3). (B) mRNA expression of RERG, IL8, IL1β and TNFα in xenografts of nude mice was determined by qRT-PCR (n = 8). GAPDH was used as an internal control. (C) IHC analyses of the expression of VEGF in tumors from nude mice. Original magnification is × 200. Bar represents 50 μm. Data are shown as means ± SD. **: P < 0.01, ***: P < 0.001 by Student’s t-test or Mann-Whitney U test. (JPG 1577 kb) [file 13046_2017_554_MOESM3_ESM.jpg]
