# Supplementary material for: RERG suppresses cell proliferation, migration and angiogenesis through ERK/NF-κB signaling pathway in nasopharyngeal carcinoma
Source: J Exp Clin Cancer Res. 2017 Jun 28;36:88. doi: 10.1186/s13046-017-0554-9 (PMC5490152; doi:10.1186/s13046-017-0554-9)
Supplement: Supplementary file 4 — Supplementary methods. (DOCX 14 kb) [file 13046_2017_554_MOESM4_ESM.docx]

**Methyl-capture sequencing**

Genomic DNA from 7 NPC biopsies and 5 NNE samples were extracted using a QIAamp DNA Mini Kit (Qiagen). DNA was fragmented to obtain the desired length of 150 bp by an ultra-sonicator (Covaris, Woburn, MA). We employed the MethylMiner Methylated DNA Enrichment Kit (Invitrogen) to select methylated DNA from 12.5 μg DNA fragments. The final two fractions of highly methylated DNA, which corresponded to gradient elution buffer concentrations of 0.6 M and 2 M NaCl, were collected. The recovered DNA in the 2 M NaCl elution buffer was purified with a PureLink PCR Purification Kit (Invitrogen). After purification, DNA samples were prepared, subjected to PCR and followed by SOLiD sequencing, that were performed by Mie University Life Science Research Center using a SOLiD System (Applied Biosystems, Foster City, CA) with mapping to the human reference genome (hg 19). Sequence data was processed using Partek Genomics Suite (Partek Incorporated, Saint Louis, MO) for further statistical analyses.

**Gene expression array analysis**

The same tissue samples as for methyl-capture sequencing were used to extract RNA by using mirVana miRNA Isolation Kit (Ambion, Carlsbad, CA, USA) according to the manufacturer's instructions. Fifty nanograms of RNA were subjected to Agilent SurePrint G3 Human GE microarray analysis (8 x 60K, 1 color, Agilent Technologies, Santa Clara, CA) for gene expression evaluation (Hokkaido System Science, Sapporo, Japan).
